# IcoSema in Weight Management and Glycaemic Control: A Systematic Review and Meta-Analysis

**DOI:** 10.7759/cureus.116875

**Published:** 2026-09-24

**Authors:** Mahmoud Ismail Nouh, Hani Raka Karrar, Hala AlButi, Rehab Salah Aldin Alhendi, Sara A Alharbi, Sarah M Alfakih, Fatehi Y Thabit, Mohammed Ameer Essaa Abdulrahman, Abdelaziz M Aman, Ghadeer M Murshid, Adel Balaish, Yousef Ismail Nouh, Mawadah Ismaeel Nooh

**Affiliations:** 1 Pharmaceutical Care Department, King Fahad Armed Forces Hospital, Jeddah, SAU; 2 Medicine and Surgery Department, Ibn Sina National College for Medical Studies, Makkah, SAU; 3 Faculty of Pharmacy, Al-Azhar University, Cairo, EGY; 4 Pharmaceutical Care Department, Dr. Samir Abbas Hospital, Jeddah, SAU; 5 Obstetrics and Gynecology Department, Ministry of Health, Makkah, SAU; 6 Pharmaceutical Care Department, King Abdulaziz University, Jeddah, SAU; 7 Pharmaceutical Care Department, Al Salama Hospital, Jeddah, SAU; 8 Pediatric Surgery Department, Ministry of National Guard Health Affairs, Jeddah, SAU; 9 Family Medicine Department, Ministry of Health, Makkah, SAU

**Keywords:** glycaemic control, icosema, insulin-icodec, semaglutide, type 2 diabetes, weight management

## Abstract

IcoSema is a once-weekly, fixed-ratio injection that combines insulin icodec, a weekly basal insulin, with semaglutide, a glucagon-like peptide-1 (GLP-1) receptor agonist. It is intended to control fasting and post-meal glucose in a single weekly injection whilst limiting the weight gain and hypoglycaemia that accompany insulin therapy. Its pivotal trials each used a different comparison treatment, and the only previous pairwise synthesis pooled them into single estimates carrying extreme statistical heterogeneity. We therefore re-synthesised the randomised evidence using a comparator-specific approach. Eight sources (PubMed/MEDLINE, Embase, Scopus, Web of Science, Cochrane Central Register of Controlled Trials (CENTRAL), Cochrane Database of Systematic Reviews (CDSR), ClinicalTrials.gov, and citation searching) were searched from inception to September 5, 2026, for randomised controlled trials of IcoSema in adults with type 2 diabetes. Two reviewers independently screened records, extracted data, and applied Cochrane Risk of Bias 2. Covariate-adjusted treatment differences were combined by random-effects inverse-variance meta-analysis, stratified by comparator class, with an exploratory all-comparator pool retained for comparison only and certainty rated with GRADE. Four phase 3 trials (3,138 participants) were eligible: COMBINE 1, 2, 3 and 4. Against basal insulin alone (COMBINE 1 and 4; two trials, 1,776 participants), IcoSema lowered HbA1c by 0.74 percentage points (95% confidence interval (CI) −0.95 to −0.53; I² = 63%), reduced body weight by 5.15 kg (95% CI −6.10 to −4.19; I² = 70%), and reduced clinically significant or severe hypoglycaemia (rate ratio 0.35, 95% CI 0.14 to 0.87; I² = 84%). Against semaglutide alone (COMBINE 2; single trial), IcoSema lowered HbA1c by a further 0.44 percentage points (95% CI −0.56 to −0.33), but body weight was 4.54 kg higher (95% CI 3.84 to 5.23), with no significant difference in hypoglycaemia. Against daily basal-bolus insulin (COMBINE 3; single trial), IcoSema met non-inferiority for HbA1c (−0.06 percentage points, 95% CI −0.22 to 0.09), reduced body weight by 6.72 kg (95% CI −7.58 to −5.86), and reduced hypoglycaemia (rate ratio 0.12, 95% CI 0.08 to 0.17). Exploratory pooling across all comparators produced estimates with I² of 92%-99.5% that should not be read as an overall treatment effect. The effects of IcoSema on glycaemia, weight, and hypoglycaemia differ in size, and for body weight in direction, according to the regimen it is compared with; comparator-specific estimates, not a single pooled figure, should inform interpretation.

## Introduction and background

Type 2 diabetes and obesity are closely linked and increasingly treated together. More than half a billion adults worldwide are living with diabetes; the burden is rising fastest in low- and middle-income countries, and excess body weight is the most important modifiable driver of new cases [1]. Treatment goals have widened accordingly: alongside durable control of blood glucose, measured in practice by glycated haemoglobin (HbA1c), clinicians are asked to limit weight gain, avoid hypoglycaemia (dangerously low blood glucose), and reduce cardiovascular risk [2]. Insulin sits awkwardly within that framework. Because insulin-producing beta cells fail progressively, many people with type 2 diabetes eventually need injected insulin. Basal insulin, a long-acting insulin given once daily or, more recently, once weekly, reliably causes weight gain and hypoglycaemia, and intensifying to a basal-bolus regimen, which adds mealtime insulin, can require up to five injections every day together with a comparable number of glucose measurements [2,3]. Burden on that scale is a recognised barrier to starting insulin, taking it as prescribed and staying on it [4,5]. Glucagon-like peptide-1 (GLP-1) receptor agonists offer a mechanistically complementary answer: they increase insulin secretion only when glucose is high, suppress glucagon, slow gastric emptying, and promote satiety, so they lower glucose and body weight with little intrinsic risk of hypoglycaemia [6,7]. Fixed-ratio combinations, in which a basal insulin and a GLP-1 receptor agonist are co-formulated in a single injection at a set ratio, address fasting hyperglycaemia and insulin-associated weight gain at the same time [8].

IcoSema extends that concept to a once-weekly product by combining insulin icodec with semaglutide. Insulin icodec is engineered for strong, reversible albumin binding and slow clearance, giving a half-life of roughly 196 hours that supports once-weekly dosing with a flat exposure profile [9]. Its glycaemic efficacy as a standalone basal insulin was established across the ONWARDS programme [10,11]. Semaglutide has well-characterised glycaemic and weight benefits [6]. Pharmacokinetic work shows that co-formulation does not meaningfully change the exposure of either component [12]. The clinical promise is a single weekly injection that lowers glucose, protects against weight gain, and reduces hypoglycaemia. Four randomised phase 3 trials, COMBINE 1 to 4, now define the evidence base [13-16]. Each used a different comparator, and the comparators are not interchangeable. COMBINE 1 compared IcoSema with once-weekly insulin icodec alone in people already using basal insulin, isolating what the semaglutide component adds to an insulin backbone [13]. COMBINE 2 compared IcoSema with once-weekly semaglutide alone in people already using a GLP-1 receptor agonist, isolating what the insulin component adds to a GLP-1 backbone [14]. COMBINE 3 compared IcoSema with a full daily basal-bolus regimen, asking whether a single weekly injection can replace the most demanding insulin schedule in routine use [15]. COMBINE 4, published in August 2026, compared IcoSema with once-daily insulin glargine U100 in insulin-naive people with poorly controlled diabetes on oral agents, the point at which insulin is usually first started [16]. These four comparisons correspond to four distinct decisions that a clinician might face, and the expected effect of IcoSema differs between them. In particular, IcoSema is expected to produce less weight loss than semaglutide alone but substantially more than any insulin comparator, so the direction of the weight effect depends on which comparison is being made. That is why a conventional meta-analysis of these trials is problematic. A previous synthesis pooled COMBINE 1 to 3 into single summary estimates and reported I² values of 95% for HbA1c and 100% for body weight, with a pooled weight estimate whose confidence interval (CI) spanned about 14 kg [17]. A network meta-analysis has since compared IcoSema indirectly with the once-daily fixed-ratio product IDegLira [18], and a further pairwise synthesis restricted to the two insulin-comparator trials has been published, excluding COMBINE 2 on the grounds that its comparator answers a different clinical question [19]. Neither approach fully resolves the underlying interpretive problem, which is that a single pooled number cannot answer several different clinical questions at once, whilst excluding trials discards randomised evidence.

We therefore conducted a systematic review and meta-analysis restricted to randomised controlled trials of IcoSema, in which comparator-specific synthesis was the prespecified primary analysis and the conventional all-comparator pool was retained only as an exploratory reference. Our aims were to estimate the effect of IcoSema on HbA1c, body weight, and hypoglycaemia separately for each class of comparator, to quantify how much of the between-trial heterogeneity is associated with comparator class, and to describe the certainty of each estimate.

## Review

Materials and methods

Protocol, Registration, and Reporting

The review was conducted according to the Cochrane Handbook [20] and is reported in accordance with the Preferred Reporting Items for Systematic Reviews and Meta-Analyses (PRISMA) 2020 statement [21]. The protocol was developed following PRISMA-P 2015 [22] and prospectively registered in PROSPERO (CRD420261404370). Three deviations from the registered protocol are declared in the Limitations section.

Eligibility Criteria

Eligibility was defined using the PICOS framework (Table 1). We included randomised controlled trials enrolling adults (aged 18 years or older) with type 2 diabetes, comparing once-weekly IcoSema at any dose with any active glucose-lowering comparator or placebo, and reporting change in HbA1c or change in body weight. Non-randomised studies, single-arm studies, pharmacokinetic studies without glycaemic or anthropometric efficacy endpoints, reviews, editorials, and preclinical work were excluded. Where several reports described the same trial, the primary publication was the index report, and secondary analyses were used only to corroborate extracted values.

**Table 1:** Eligibility criteria defined by the PICOS framework CGM: continuous glucose monitoring

Domain	Inclusion criteria	Exclusion criteria
Population	Adults aged ≥18 years with a diagnosis of type 2 diabetes mellitus, irrespective of background glucose-lowering therapy, sex, ethnicity, or geographical region	Type 1 diabetes; gestational diabetes; paediatric populations; healthy volunteers
Intervention	Once-weekly IcoSema (fixed-ratio combination of insulin icodec and semaglutide) at any dose, administered subcutaneously	Sequential or free-combination administration of insulin icodec and semaglutide as separate injections
Comparator	Any active glucose-lowering comparator (once-weekly or once-daily basal insulin, once-weekly semaglutide, basal-bolus insulin, other fixed-ratio combinations) or placebo	Comparison against another IcoSema dose only, with no external comparator arm
Outcomes	At minimum, change in HbA1c or change in body weight from baseline. Secondary: HbA1c target attainment, fasting plasma glucose, CGM time in range, level 2/3 hypoglycaemia, insulin dose, adverse events, discontinuation	Studies reporting neither co-primary outcome; pharmacokinetic or pharmacodynamic endpoints only
Study design	Randomised controlled trials (parallel-group or crossover), any phase, any duration	Non-randomised studies; single-arm studies; observational studies; modelling studies; narrative reviews; editorials; conference abstracts superseded by a full report; preclinical work
Other limits	Published from database inception to September 5, 2026	-

Information Sources and Search Strategy

We searched PubMed/MEDLINE, Embase, Scopus, Web of Science Core Collection, the Cochrane Central Register of Controlled Trials (CENTRAL), the Cochrane Database of Systematic Reviews (CDSR), and ClinicalTrials.gov from database inception. The original search was run on June 30, 2026. In response to peer review, the complete search was re-executed on September 5, 2026, across all sources, and the WHO International Clinical Trials Registry Platform was additionally checked for unpublished or ongoing trials. Reference lists of all included reports, of previous syntheses, and of relevant narrative reviews were hand-searched. No language filter was applied at the search stage. The core PubMed string was (“IcoSema” OR “NNC0487-0111” OR (“insulin icodec” AND “semaglutide”) OR “icodec/semaglutide”) AND (“diabetes mellitus, type 2”[MeSH] OR “type 2 diabetes” OR T2DM) AND (randomized controlled trial[pt] OR randomised OR randomized OR COMBINE). Database-specific strategies and yields are given in Table 2.

**Table 2:** Full database-specific search strategies and yields CENTRAL: Central Register of Controlled Trials; CDSR: Cochrane Database of Systematic Reviews

#	Source	Interface/segment	Search string	Records
1	PubMed/MEDLINE	pubmed.ncbi.nlm.nih.gov	(“IcoSema”[tiab] OR “NNC0487-0111”[tiab] OR (“insulin icodec”[tiab] AND “semaglutide”[tiab]) OR “icodec/semaglutide”[tiab]) AND (“Diabetes Mellitus, Type 2”[MeSH] OR “type 2 diabetes”[tiab] OR “T2DM”[tiab]) AND (randomized controlled trial[pt] OR randomised[tiab] OR randomized[tiab] OR “COMBINE”[tiab])	84
2	Embase	Ovid, 1974-2026	(icosema OR “NNC0487 0111” OR (insulin icodec AND semaglutide)).mp. AND (exp non insulin dependent diabetes mellitus/ OR “type 2 diabetes”.mp.) AND (randomized controlled trial/ OR random$.mp.)	103
3	Scopus	Elsevier	TITLE-ABS-KEY(icosema OR “NNC0487-0111” OR (“insulin icodec” AND semaglutide)) AND TITLE-ABS-KEY(“type 2 diabetes” OR t2dm) AND TITLE-ABS-KEY(random*)	77
4	Web of Science Core Collection	Clarivate	TS=(icosema OR “NNC0487-0111” OR (“insulin icodec” AND semaglutide)) AND TS=(“type 2 diabetes” OR t2dm) AND TS=(random*)	59
5	Cochrane CENTRAL	Cochrane Library	(icosema OR (“insulin icodec” AND semaglutide)):ti,ab,kw AND (“type 2 diabetes”):ti,ab,kw —Trials	25
6	CDSR	Cochrane Library	(icosema OR (“insulin icodec” AND semaglutide)):ti,ab,kw — Reviews and Protocols	2
7	ClinicalTrials.gov	Intervention/treatment search	IcoSema OR (insulin icodec AND semaglutide); condition: type 2 diabetes; study type: interventional	15
8	Citation searching	Reference lists of included reports and previous syntheses	-	7
Total identified				372
Duplicates removed				213
Unique records screened				159

Study Selection

Records were exported to reference-management software, deduplicated automatically, and then checked manually. Two reviewers (M.I.N. and H.R.K.) independently screened all titles and abstracts against the eligibility criteria, then independently assessed every full text retrieved. Agreement at each stage was recorded, and disagreements were resolved by discussion; a third reviewer (H.A.) adjudicated when consensus could not be reached. Reasons for exclusion at the full-text stage were documented and are shown in the PRISMA flow diagram (Figure 1).

**Figure 1:**
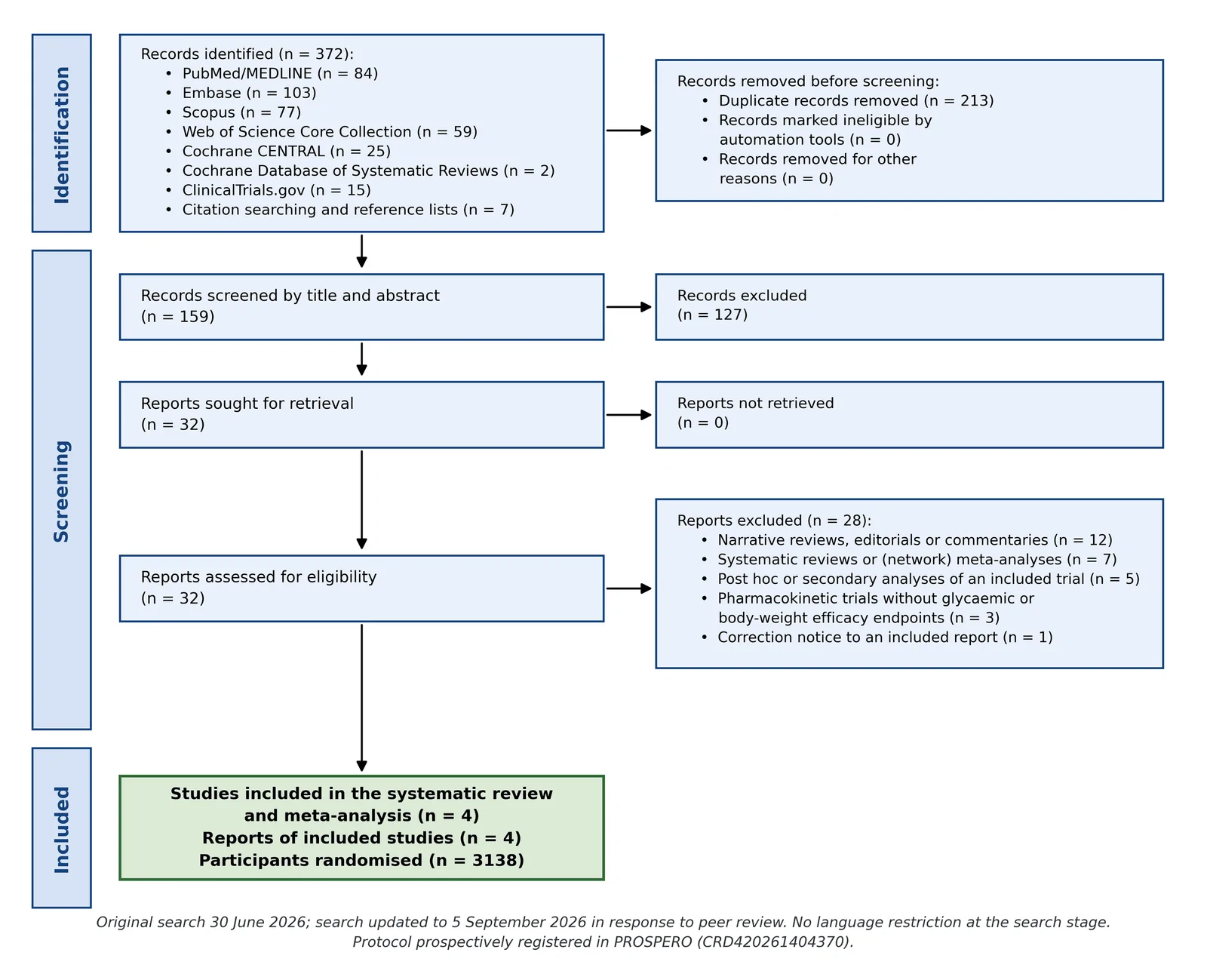
PRISMA 2020 flow diagram PRISMA: Preferred Reporting Items for Systematic Reviews and Meta-Analyses; CENTRAL: Central Register of Controlled Trials

Data Extraction and Outcomes

A piloted extraction form captured trial identifiers and registration numbers, design, setting, number of sites and countries, randomisation ratio, sample size, participant baseline characteristics, intervention, and comparator specification including titration algorithm, follow-up duration, funding source, and all prespecified outcomes. The same two reviewers extracted data independently into separate copies of the form; the copies were compared field by field, and every discrepancy was resolved against the source publication and, where relevant, the trial registry entry. Consistent with the registered protocol, trial investigators and sponsors were not contacted for unpublished data; outcomes that could not be extracted from the public record are reported as such. Co-primary outcomes were change in HbA1c (percentage points) and change in body weight (kg), both from baseline to end of treatment. Secondary outcomes were the rate of clinically significant (level 2, glucose below 3.0 mmol/L) or severe (level 3) hypoglycaemia, defined per the International Hypoglycaemia Study Group [23]; attainment of HbA1c below 7.0%; change in fasting plasma glucose; continuous glucose monitoring time in range 3.9-10.0 mmol/L [24]; weekly total insulin dose; serious adverse events; and treatment discontinuation due to adverse events.

Risk-of-Bias Assessment

Two reviewers independently appraised each trial with version 2 of the Cochrane risk-of-bias tool for randomised trials (RoB 2), across randomisation process, deviations from intended interventions, missing outcome data, measurement of the outcome, and selection of the reported result [25]. Judgements were made against the treatment-policy estimand for the co-primary outcomes. Domain-level and overall judgements were rendered graphically following the robvis approach [26]. Justifications are given in Table 3.

**Table 3:** Cochrane RoB 2 domain-level judgements with supporting justification

Domain	COMBINE 1 [13]	COMBINE 2 [14]	COMBINE 3 [15]	COMBINE 4 [16]
D1. Randomisation process	Low. 1:1 allocation via a validated randomisation and trial supply management system; allocation concealed until randomisation; baseline characteristics balanced	Low. 1:1 allocation via central interactive web-response system; no baseline imbalance suggesting a failure of randomisation	Low. 1:1 allocation via an automated, interactive central web-response system; balanced baseline characteristics	Low. 1:1 central allocation without stratification; baseline HbA1c and demographics balanced between arms
D2. Deviations from intended interventions	Some concerns. Open-label design; treat-to-target titration by unmasked investigators. Treatment-policy estimand in the full analysis set mitigates but does not eliminate the concern	Some concerns. Open-label design; participants and investigators aware of assignment; treatment-policy estimand applied to the full analysis set	Some concerns. Open-label design with a marked asymmetry in injection frequency (1 per week vs. up to 35 per week); unmasked titration of both basal and bolus components	Some concerns. Open-label design; both arms titrated to a fasting glucose target of 3.9-5.0 mmol/L by unmasked investigators; treatment-policy estimand
D3. Missing outcome data	Low. Outcome data available for the great majority of randomised participants; missing data handled under a treatment-policy estimand with multiple imputation	Low. 93.6% (IcoSema) and 96.2% (semaglutide) of participants completed week 52 without permanent treatment discontinuation	Low. Retention high in both arms; treatment-policy estimand with prespecified imputation	Low. All 485 randomised participants analysed under the treatment-policy estimand; retention high in both arms
D4. Measurement of the outcome	Low. HbA1c by central laboratory; body weight on calibrated scales; hypoglycaemia confirmed by blood glucose meter	Low. Central laboratory HbA1c; objective anthropometry; biochemically confirmed hypoglycaemia	Low. Central laboratory HbA1c; objective anthropometry; hypoglycaemia confirmed at <3.0 mmol/L by meter	Low. Central laboratory HbA1c; objective anthropometry; hypoglycaemia confirmed at <3.0 mmol/L by meter
D5. Selection of the reported result	Low. Protocol and statistical analysis plan public; prespecified hierarchical testing sequence followed; all confirmatory endpoints reported	Low. Registered protocol; prespecified estimands and confirmatory endpoints reported as planned	Low. Registered protocol with prespecified non-inferiority margin and hierarchical testing of confirmatory secondary endpoints	Low. Registered protocol (NCT06269107); primary and confirmatory secondary endpoints reported as prespecified
Overall	Some concerns	Some concerns	Some concerns	Some concerns

Risk of bias due to missing results was assessed at the outcome level by comparing each trial’s registry entry and published statistical analysis plan against the outcomes reported, and by checking whether any prespecified outcome was absent from the primary publication. Small-study effects could not be evaluated statistically, because fewer than 10 trials contributed to any outcome; this is addressed qualitatively in the Limitations section.

Data Synthesis

All included trials reported outcomes as estimated treatment differences from analysis-of-covariance or negative-binomial models adjusted for baseline value and region. We therefore used the generic inverse-variance method throughout, which preserves the trials’ own covariate adjustment. Standard errors were recovered from published 95% CIs as (upper − lower)/3.92. Continuous outcomes were combined as mean differences and hypoglycaemia rates as rate ratios on the natural-logarithm scale. All syntheses used the DerSimonian-Laird random-effects model [27], prespecified because clinical and methodological diversity was anticipated. The effect estimates and standard errors entered into the analysis are listed in Table 4. The primary analysis was stratified by comparator class. Three strata were formed: IcoSema versus basal insulin alone (COMBINE 1, comparator once-weekly icodec; COMBINE 4, comparator once-daily glargine U100), IcoSema versus a GLP-1 receptor agonist alone (COMBINE 2), and IcoSema versus daily basal-bolus insulin (COMBINE 3). The basal-insulin stratum contains two trials and therefore yields a meta-analytic estimate with an estimable I²; the other two strata each contain a single trial, and their estimates are the trial’s own covariate-adjusted results presented within the review framework rather than pooled estimates. We use the term “comparator-specific estimate” for all three and specify the number of contributing trials wherever an estimate is reported. An all-comparator pooled estimate was also computed, for comparability with the earlier synthesis. This estimate is labelled exploratory throughout and is not interpreted as an overall treatment effect of IcoSema. Differences between strata were tested with the χ² test for subgroup differences. Because comparator class and individual trial are completely confounded in the single-trial strata, and because the trials also differ in population and duration, a significant subgroup test indicates that between-trial differences are consistent with, but cannot be attributed causally to, comparator class. Heterogeneity was quantified with τ², the χ² test, and I², interpreted using the conventional 25%/50%/75% thresholds [28]. Prespecified sensitivity analyses included a leave-one-out analysis of the exploratory pool and exclusion of the trial with an imputed standard error (below). In response to peer review, we added three further sensitivity analyses: a four-stratum analysis in which once-weekly and once-daily basal insulin comparators were kept separate, re-estimation of the two-trial basal-insulin stratum with the Hartung-Knapp adjustment [29], and a fixed-effect estimate for that stratum. With only two to four trials, the DerSimonian-Laird estimate of τ² is imprecise, and the resulting intervals may be anticonservative, which is a further reason for treating the exploratory pool as a reference rather than a result. Funnel-plot asymmetry and Egger’s test [30] were not performed because fewer than 10 trials contributed to any outcome, in line with Cochrane Handbook guidance [20]. Analyses were performed in Review Manager (RevMan) 8.x (The Cochrane Collaboration, London, UK) and replicated independently in Python 3.11 (SciPy 1.11) (Python Software Foundation, Wilmington, DE, USA); forest plots follow the RevMan layout. One value required imputation. The CI around the body-weight treatment difference in COMBINE 1 is not reported in the public record, although the point estimate is. Following Cochrane Handbook guidance on borrowing dispersion parameters from comparable studies within the same synthesis [20], we derived the residual standard deviation implied by each of the other 52-week trials and applied the larger of the two (5.72 kg), which yields the more conservative interval. A sensitivity analysis omitting COMBINE 1 is reported in the Results section.

**Table 4:** Effect estimates and derived SEs used in the meta-analysis ^a^Imputed value: the 95% CI for the body-weight treatment difference in COMBINE 1 is not reported in the public record. The SE was derived by borrowing the larger residual SD (5.72 kg) implied by the other 52-week trials (COMBINE 3 and 4), following Cochrane Handbook guidance [20]; the CI shown was back-calculated from this SE. See the sensitivity analysis omitting COMBINE 1. GLP-1: glucagon-like peptide-1; SE: standard error; CI: confidence interval; SD: standard deviation

Outcome	Trial	Comparator stratum	Effect estimate	95% CI	Analysis-scale value	Derived SE	n (IcoSema)	n (comparator)
HbA1c (%-points)	COMBINE 1 [13]	Basal insulin alone (once-weekly icodec)	−0.66	−0.76 to −0.57	−0.6600	0.0485	646	645
	COMBINE 4 [16]	Basal insulin alone (once-daily glargine U100)	−0.88	−1.12 to −0.63	−0.8800	0.1250	243	242
	COMBINE 2 [14]	GLP-1 receptor agonist alone	−0.44	−0.56 to −0.33	−0.4400	0.0587	342	341
	COMBINE 3 [15]	Daily basal-bolus insulin	−0.06	−0.22 to 0.09	−0.0600	0.0791	340	339
Body weight (kg)	COMBINE 1 [13]	Basal insulin alone (once-weekly icodec)	−5.59	−6.21 to −4.97^a^	−5.5900	0.3182^a^	646	645
	COMBINE 4 [16]	Basal insulin alone (once-daily glargine U100)	−4.61	−5.46 to −3.75	−4.6100	0.4362	243	242
	COMBINE 2 [14]	GLP-1 receptor agonist alone	+4.54	3.84 to 5.23	4.5400	0.3546	342	341
	COMBINE 3 [15]	Daily basal-bolus insulin	−6.72	−7.58 to −5.86	−6.7200	0.4388	340	339
Level 2/3 hypoglycaemia, rate ratio	COMBINE 1 [13]	Basal insulin alone (once-weekly icodec)	0.22	0.14 to 0.36	−1.5141	0.2409	646	645
	COMBINE 4 [16]	Basal insulin alone (once-daily glargine U100)	0.56	0.32 to 0.97	−0.5798	0.2829	243	242
	COMBINE 2 [14]	GLP-1 receptor agonist alone	1.20	0.53 to 2.69	0.1823	0.4144	342	341
	COMBINE 3 [15]	Daily basal-bolus insulin	0.12	0.08 to 0.17	−2.1203	0.1923	340	339

Certainty of Evidence

Two reviewers rated the certainty of evidence for each outcome and each comparator stratum using GRADE, considering risk of bias, inconsistency, indirectness, imprecision, and publication bias [31,32]. For single-trial strata, inconsistency was rated as not assessable rather than absent.

Results

Study Selection

The updated search returned 372 records, of which 213 were duplicates. Of the 159 unique records screened, 127 were excluded at title and abstract. Thirty-two full-text reports were assessed, and 28 were excluded, most commonly because they were narrative reviews or commentaries (n = 12), previous evidence syntheses (n = 7), or post hoc analyses of an already included trial (n = 5). Four randomised controlled trials met all eligibility criteria (Figure 1). COMBINE 4 was the only eligible trial identified by the updated search; no further completed or ongoing randomised trial of IcoSema with a glycaemic or body-weight endpoint was found in ClinicalTrials.gov or the WHO registry platform as of September 5, 2026.

Characteristics of Included Trials

The four trials randomised 3,138 adults with type 2 diabetes and were sponsored by the same manufacturer; all were open-label, used 1:1 allocation and multinational recruitment, and analysed the primary endpoint in the full analysis set under a treatment-policy estimand (Table 5) [13-16]. Where they diverge is in the clinical question each was built to answer.

**Table 5:** Characteristics of the four included randomised controlled trials GLP-1: glucagon-like peptide-1

Characteristic	COMBINE 1 [13]	COMBINE 2 [14]	COMBINE 3 [15]	COMBINE 4 [16]
First author, year	Mathieu, 2025	Lingvay, 2025	Billings, 2025	Ji, 2026
Registration	NCT05352815	NCT05259033	NCT05013229	NCT06269107
Design	Open-label, treat-to-target, randomised, phase 3a; superiority	Open-label, randomised, phase 3a; IcoSema titrated to a fasting glucose target	Open-label, treat-to-target, randomised, phase 3a; non-inferiority (0.3 %-point margin)	Open-label, treat-to-target, randomised, phase 3b; superiority
Sites/countries	192 sites, 20 countries and regions	121 sites, 13 countries	109 sites, 14 countries	97 sites, 9 countries
Randomised (n)	1,291	683	679	485
Intervention arm (n)	IcoSema (646)	IcoSema (342)	IcoSema (340)	IcoSema (243)
Comparator arm (n)	Insulin icodec once weekly (645)	Semaglutide 1.0 mg once weekly (341)	Insulin glargine U100 once daily + insulin aspart 2-4 times daily (339)	Insulin glargine U100 once daily (242)
Comparator stratum	Basal insulin alone	GLP-1 receptor agonist alone	Daily basal-bolus insulin	Basal insulin alone
Population at entry	Daily basal insulin ± oral agents	GLP-1 receptor agonist	Daily basal insulin 20-80 U/day	Insulin-naive; oral glucose-lowering medication; HbA1c ≥ 8.0%
Treatment duration	52 weeks (safety follow-up to week 57)	52 weeks (safety follow-up to week 57)	52 weeks (safety follow-up to week 57)	40 weeks (safety follow-up to week 45)
Primary endpoint	Change in HbA1c, baseline to week 52	Change in HbA1c, baseline to week 52	Change in HbA1c, baseline to week 52	Change in HbA1c, baseline to week 40
Analysis population	Full analysis set; treatment-policy estimand	Full analysis set; treatment-policy estimand	Full analysis set; treatment-policy estimand	Full analysis set; treatment-policy estimand
Funding	Novo Nordisk	Novo Nordisk	Novo Nordisk	Novo Nordisk

COMBINE 1 recruited 1,291 participants already receiving daily basal insulin across 192 sites in 20 countries and regions, and compared IcoSema with once-weekly insulin icodec alone over 52 weeks in a treat-to-target superiority design [13]. COMBINE 2 recruited 683 participants inadequately controlled on a GLP-1 receptor agonist across 121 sites in 13 countries and compared IcoSema with once-weekly semaglutide 1.0 mg over 52 weeks [14]. COMBINE 3 recruited 679 participants receiving 20-80 U of daily basal insulin across 109 sites in 14 countries and tested IcoSema against a basal-bolus regimen (insulin glargine U100 plus two to four daily aspart injections) over 52 weeks for non-inferiority within a 0.3 percentage-point margin [15]. COMBINE 4 recruited 485 insulin-naive participants with HbA1c of 8.0% or higher on oral glucose-lowering medication across 97 sites in nine countries, and compared IcoSema with once-daily insulin glargine U100 over 40 weeks in a treat-to-target superiority design, with both arms titrated to a fasting glucose target of 3.9-5.0 mmol/L [16].

Baseline characteristics are given in Table 6. The insulin-experienced trials enrolled participants with mean baseline HbA1c between 7.9% and 8.2%, whereas COMBINE 4 enrolled a population with substantially poorer control (mean baseline HbA1c 9.57% with IcoSema and 9.50% with glargine) and a median age of 58 years [16]. The outcome data extracted from each trial are set out in Table 7.

**Table 6:** Baseline characteristics of participants in the included trials Values are for the total randomised population unless stated otherwise. BMI: body mass index; GLP-1: glucagon-like peptide-1; SD: standard deviation

Characteristic	COMBINE 1 (n = 1,291)	COMBINE 2 (n = 683)	COMBINE 3 (n = 679)	COMBINE 4 (n = 485)
Age (years)	Mean 60.6 (SD 10.3)	Not reported by arm	Mean 59.6 (SD 10.4)	Median 58 (range 26-82)
Male, n (%)	799 (62)	Not reported by arm	399 (59)	286 (59)
Female, n (%)	492 (38)	Not reported by arm	280 (41)	199 (41)
Baseline HbA1c (%), mean (SD)	8.22 (0.83)	IcoSema 8.07 (0.76); semaglutide 7.93 (0.73)	Not reported by arm	IcoSema 9.57; glargine 9.50
Baseline HbA1c (mmol/mol), mean (SD)	66.3 (9.1)	IcoSema 64.7 (8.3); semaglutide 63.2 (8.0)	Not reported by arm	Not reported in abstract
Baseline body weight (kg), mean	Not reported	IcoSema 87.6; semaglutide 90.8	Not reported	Not reported in abstract
BMI (kg/m²), mean (SD)	Not reported (eligibility ceiling ≤ 40)	31.1 (4.7)	Not reported	Not reported in abstract
Diabetes duration (years), mean (SD)	Not reported	IcoSema 12.94 (6.82); semaglutide 12.34 (6.97)	Not reported	Not reported in abstract
Eligibility HbA1c range	7.0%-10.0%	Inadequate control on a GLP-1 receptor agonist	7.0%-10.0%	≥8.0%
Background therapy at entry	Daily basal insulin ± oral agents	GLP-1 receptor agonist	Daily basal insulin 20-80 U/day	Oral glucose-lowering medication (insulin-naive)

**Table 7:** Outcome data extracted from each included trial ^a^CI derived by the imputation described in the Materials and Methods section. AE: adverse event; CGM: continuous glucose monitoring; CI: confidence interval; EOR: estimated odds ratio; ETD: estimated treatment difference (IcoSema minus comparator); PYE: person-years of exposure; SE: standard error; SOC: system organ class

Outcome	COMBINE 1 (vs. icodec) [13]	COMBINE 2 (vs. semaglutide) [14]	COMBINE 3 (vs. basal-bolus) [15]	COMBINE 4 (vs. glargine U100) [16]
Change in HbA1c, IcoSema arm	−1.55 %-points (SE 0.03)	−1.35 %-points	−1.47 %-points (SE 0.05)	−3.32 %-points
Change in HbA1c, comparator arm	−0.89 %-points (SE 0.03)	−0.90 %-points	−1.40 %-points (SE 0.06)	−2.44 %-points
ETD for HbA1c (95% CI)	−0.66 (−0.76 to −0.57)	−0.44 (−0.56 to −0.33)	−0.06 (−0.22 to 0.09)	−0.88 (−1.12 to −0.63)
Change in body weight, IcoSema arm	−3.7 kg	+0.84 kg	−3.56 kg	−0.79 kg
Change in body weight, comparator arm	+1.9 kg	−3.70 kg	+3.16 kg	+3.81 kg
ETD for body weight (95% CI)	−5.59 (−6.21 to −4.97)^a^	+4.54 (3.84 to 5.23)	−6.72 (−7.58 to −5.86)	−4.61 (−5.46 to −3.75)
HbA1c < 7.0% at end of treatment	Not reported in primary publication	73.5% vs. 48.0%; EOR 3.01 (2.14 to 4.24)	Not reported in primary publication	Not reported in abstract
Change in fasting plasma glucose	Not reported in primary publication	ETD −1.07 mmol/L (−1.37 to −0.76)	Not reported in primary publication	Not reported in abstract
CGM time in range 3.9-10.0 mmol/L	75.9% vs. 61.9%	Not reported	Not reported in primary publication; postprandial glucose comparable in a post hoc analysis [33]	Not reported in abstract
Weekly total insulin dose	Not reported in primary publication	Not applicable	ETD −270 U (−303 to −236)	Not reported in abstract
Level 2 or 3 hypoglycaemia, episodes/PYE	0.14 vs. 0.63	0.042 vs. 0.036	0.21 vs. 2.23	0.29 vs. 0.59
Estimated rate ratio (95% CI)	0.22 (0.14 to 0.36)	1.20 (0.53 to 2.69)	0.12 (0.08 to 0.17)	0.56 (0.32 to 0.97)
Gastrointestinal disorders (SOC)	303/644 (47%) vs. not reported	107/341 (31.4%) vs. 117/340 (34.4%)	148/340 (44%) vs. not reported	Most frequent AE class with IcoSema; counts not in abstract
Serious adverse event, n (%)	59/644 (9) vs. 69/644 (11)	38/341 (11.1) vs. 21/340 (6.2)	Not reported	Not reported in abstract
Discontinuation due to adverse event	Not reported	2.3% vs. 2.6%	Not reported	Not reported in abstract

Risk of Bias

All four trials were judged to be at low risk of bias for the randomisation process, missing outcome data, measurement of the outcome, and selection of the reported result (Figures 2, 3; Table 3). Each nonetheless raised some concerns in the domain of deviations from intended interventions, which determined an overall judgement of some concerns for all four. The reason is intrinsic to the question being asked: insulin regimens cannot be masked when one arm receives a single weekly injection and the other receives daily injections, and in every trial the IcoSema dose was titrated by unmasked investigators against a fasting glucose target. We considered this unlikely to have materially biased HbA1c or body weight, both of which are objectively measured, though it remains a genuine limitation for patient-reported and clinician-adjudicated endpoints. Comparison of registry entries with published reports identified no prespecified confirmatory endpoint that was omitted from any primary publication.

**Figure 2:**
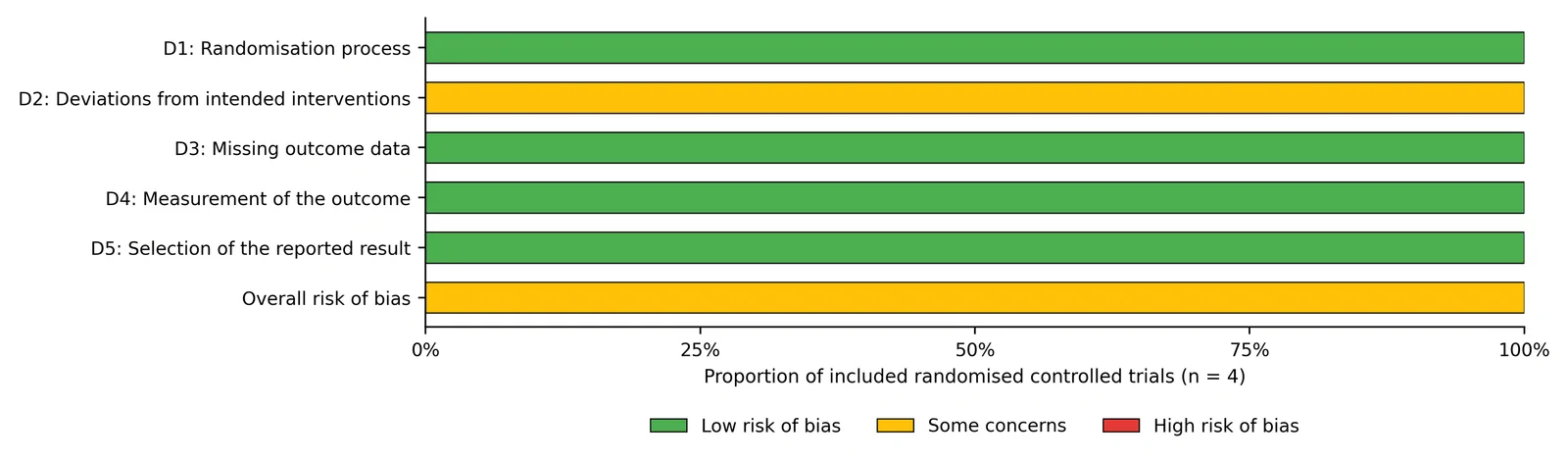
Risk-of-bias assessment graph of the included trials using the Cochrane RoB 2 tool

**Figure 3:**
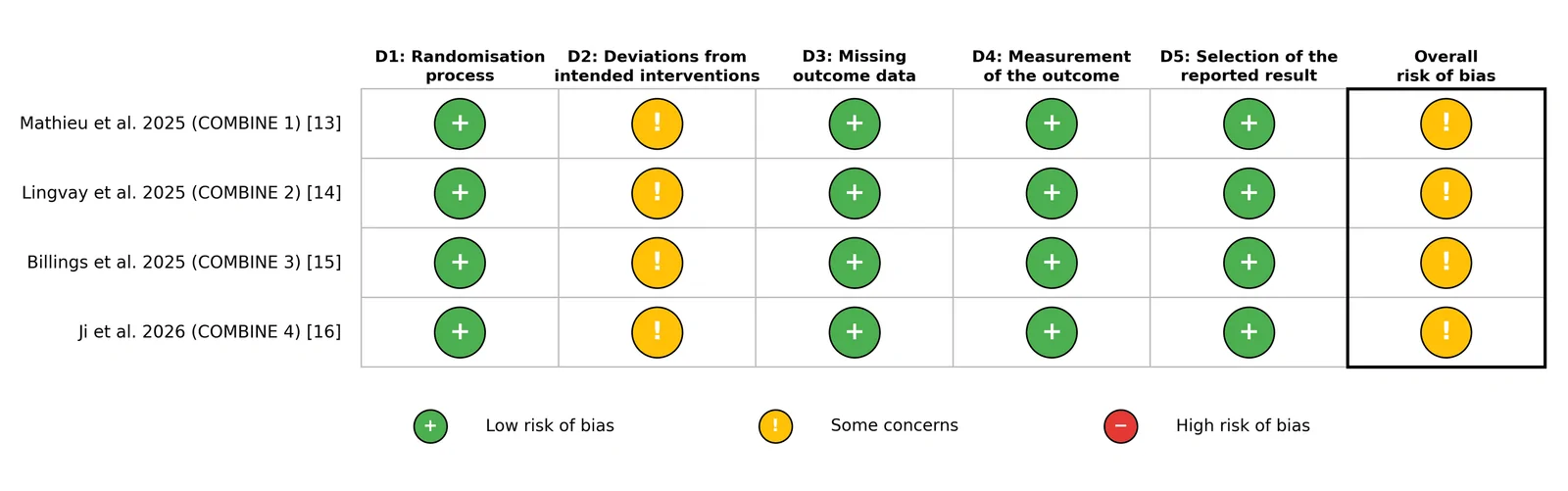
Risk-of-bias assessment summary of the included trials using the Cochrane RoB 2 tool

Glycaemic Control

Comparator-specific results are shown in Figure 4. Against basal insulin alone, the two-trial meta-analytic estimate was a reduction in HbA1c of 0.74 percentage points with IcoSema (95% CI −0.95 to −0.53; Z = 6.99, P < 0.00001), with moderate heterogeneity between COMBINE 1 and COMBINE 4 (τ² = 0.02; χ² = 2.69, df = 1, P = 0.10; I² = 62.9%). The two trials were directionally consistent: the treatment difference was −0.66 percentage points (95% CI −0.76 to −0.57) in insulin-experienced participants with a baseline HbA1c of 8.2% over 52 weeks [13], and −0.88 percentage points (95% CI −1.12 to −0.63) in insulin-naive participants with a baseline HbA1c of 9.5% over 40 weeks [16]. Against once-weekly semaglutide 1.0 mg, the single-trial estimate was a reduction of 0.44 percentage points (95% CI −0.56 to −0.33; P < 0.00001), with 73.5% versus 48.0% of participants reaching HbA1c below 7.0% (estimated odds ratio 3.01, 95% CI 2.14 to 4.24) [14]. Against daily basal-bolus therapy, the single-trial estimate was −0.06 percentage points (95% CI −0.22 to 0.09), within the prespecified 0.3 percentage-point non-inferiority margin [15]. The exploratory all-comparator pool gave a mean difference of −0.50 percentage points (95% CI −0.79 to −0.22; Z = 3.44, P = 0.0006) with τ² = 0.08, χ² = 52.12 (df = 3, P < 0.00001) and I² = 94.2%. The test for subgroup differences between the three strata was highly significant (χ² = 28.99, df = 2, P < 0.00001, I² = 93.1%).

**Figure 4:**
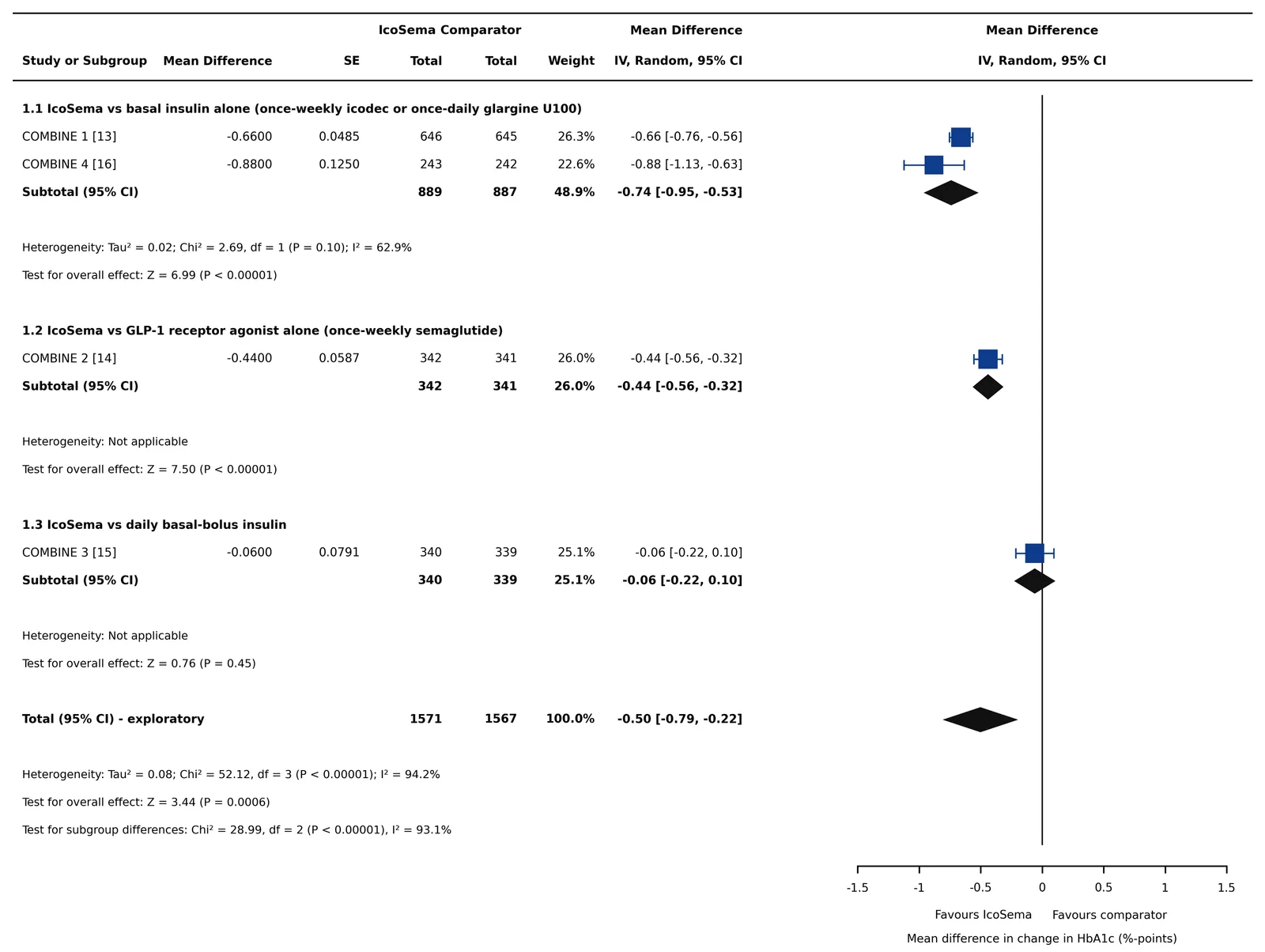
Forest plot of the mean difference in change in HbA1c CI: confidence interval; GLP-1: glucagon-like peptide-1; SE: standard error

Body Weight

Comparator-specific results are shown in Figure 5. Against basal insulin alone, the two-trial estimate was a reduction of 5.15 kg with IcoSema (95% CI −6.10 to −4.19; Z = 10.55, P < 0.00001; τ² = 0.33; χ² = 3.29, df = 1, P = 0.07; I² = 69.6%), reflecting differences of −5.59 kg versus icodec (a fall of 3.7 kg against a gain of 1.9 kg) [13] and −4.61 kg versus glargine U100 (a fall of 0.79 kg against a gain of 3.81 kg) [16]. Against daily basal-bolus therapy, the single-trial estimate was −6.72 kg (95% CI −7.58 to −5.86; P < 0.00001), reflecting a fall of 3.56 kg against a gain of 3.16 kg [15]. Against semaglutide alone, body weight was 4.54 kg higher with IcoSema (95% CI 3.84 to 5.23; P < 0.00001), reflecting a gain of 0.84 kg against a loss of 3.70 kg [14]. The direction of the weight effect therefore reverses with the comparator, and the reversal is predictable from pharmacology. Adding semaglutide to an insulin backbone offsets insulin-associated weight gain, whereas adding insulin to a semaglutide backbone partially offsets GLP-1-mediated weight loss. The exploratory all-comparator pool was −3.09 kg (95% CI −8.39 to 2.20; Z = 1.15, P = 0.25), with τ² = 29.01, χ² = 602.34 (df = 3, P < 0.00001) and I² = 99.5%. The interval is more than 10 kg wide, includes zero, and encompasses both a large weight loss and a large weight gain; it is arithmetically correct but conveys nothing a clinician could act on. The test for subgroup differences was χ² = 485.68 (df = 2, P < 0.00001, I² = 99.6%).

**Figure 5:**
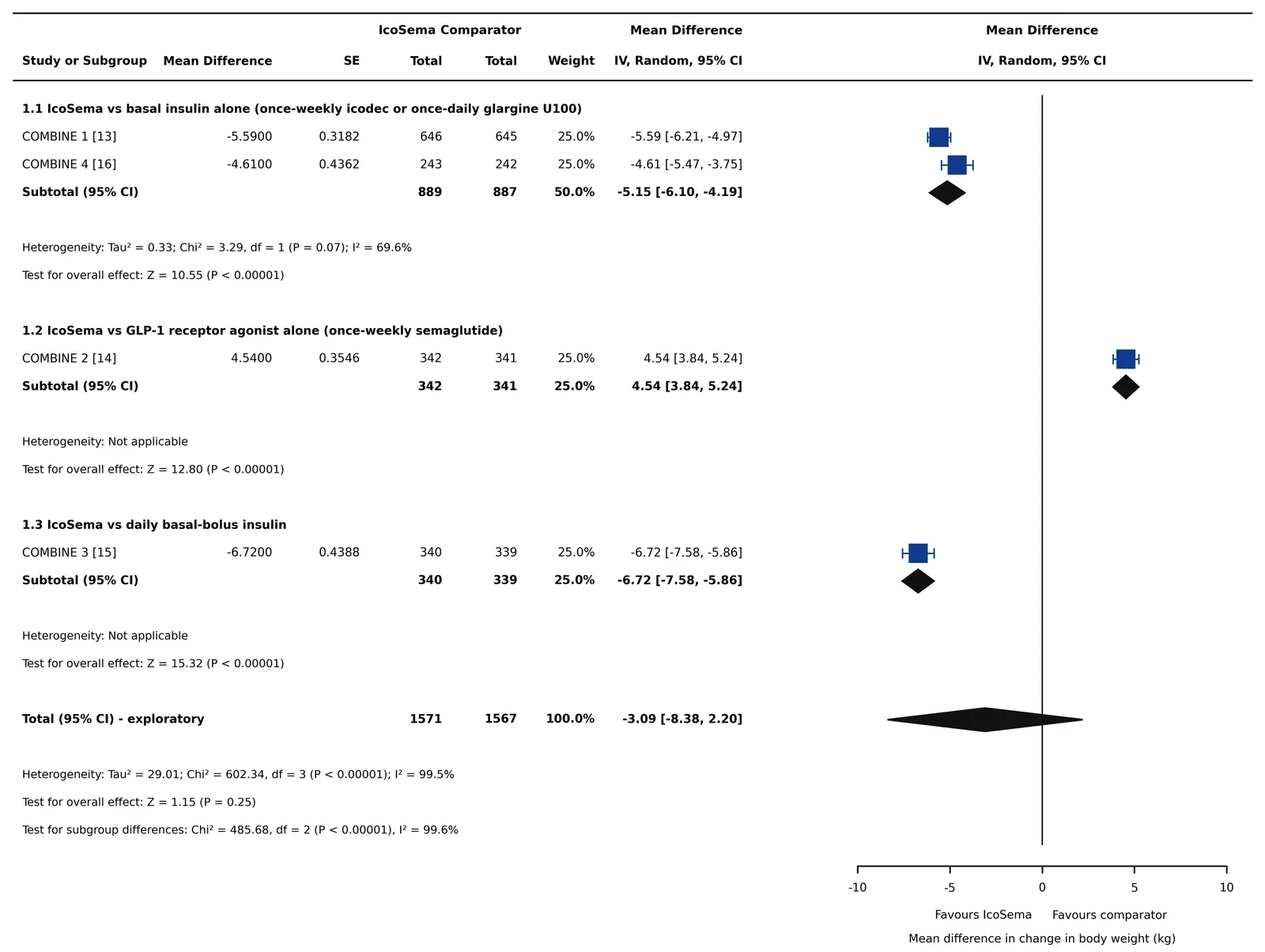
Forest plot of the mean difference in change in body weight CI: confidence interval; GLP-1: glucagon-like peptide-1; SE: standard error

Hypoglycaemia

Comparator-specific results are shown in Figure 6. Against basal insulin alone, the two-trial estimate was a rate ratio for clinically significant or severe hypoglycaemia of 0.35 (95% CI 0.14 to 0.87; Z = 2.27, P = 0.02), with substantial heterogeneity (τ² = 0.37; χ² = 6.32, df = 1, P = 0.012; I² = 84.2%): the reduction was larger versus icodec (rate ratio 0.22, 95% CI 0.14 to 0.36; 0.14 versus 0.63 episodes per person-year) [13] than versus glargine U100 (rate ratio 0.56, 95% CI 0.32 to 0.97; 0.29 versus 0.59 episodes per person-year) [16]. Against daily basal-bolus therapy, the single-trial rate ratio was 0.12 (95% CI 0.08 to 0.17; 0.21 versus 2.23 episodes per person-year), so the comparator arm accrued more than 10 times the rate seen with IcoSema, and the relative reduction translates into roughly two avoided episodes per person-year [15]. Against semaglutide alone, where the comparator arm carried no exogenous insulin, rates were low in both groups with no significant difference, although the interval remained compatible with a substantial relative increase (rate ratio 1.20, 95% CI 0.53 to 2.69; 0.042 versus 0.036 episodes per person-year); no severe episodes occurred in either arm [14]. The exploratory all-comparator pool gave a rate ratio of 0.35 (95% CI 0.14 to 0.87; Z = 2.25, P = 0.02) with I² = 91.9%; the test for subgroup differences was χ² = 27.12 (df = 2, P < 0.00001, I² = 92.6%).

**Figure 6:**
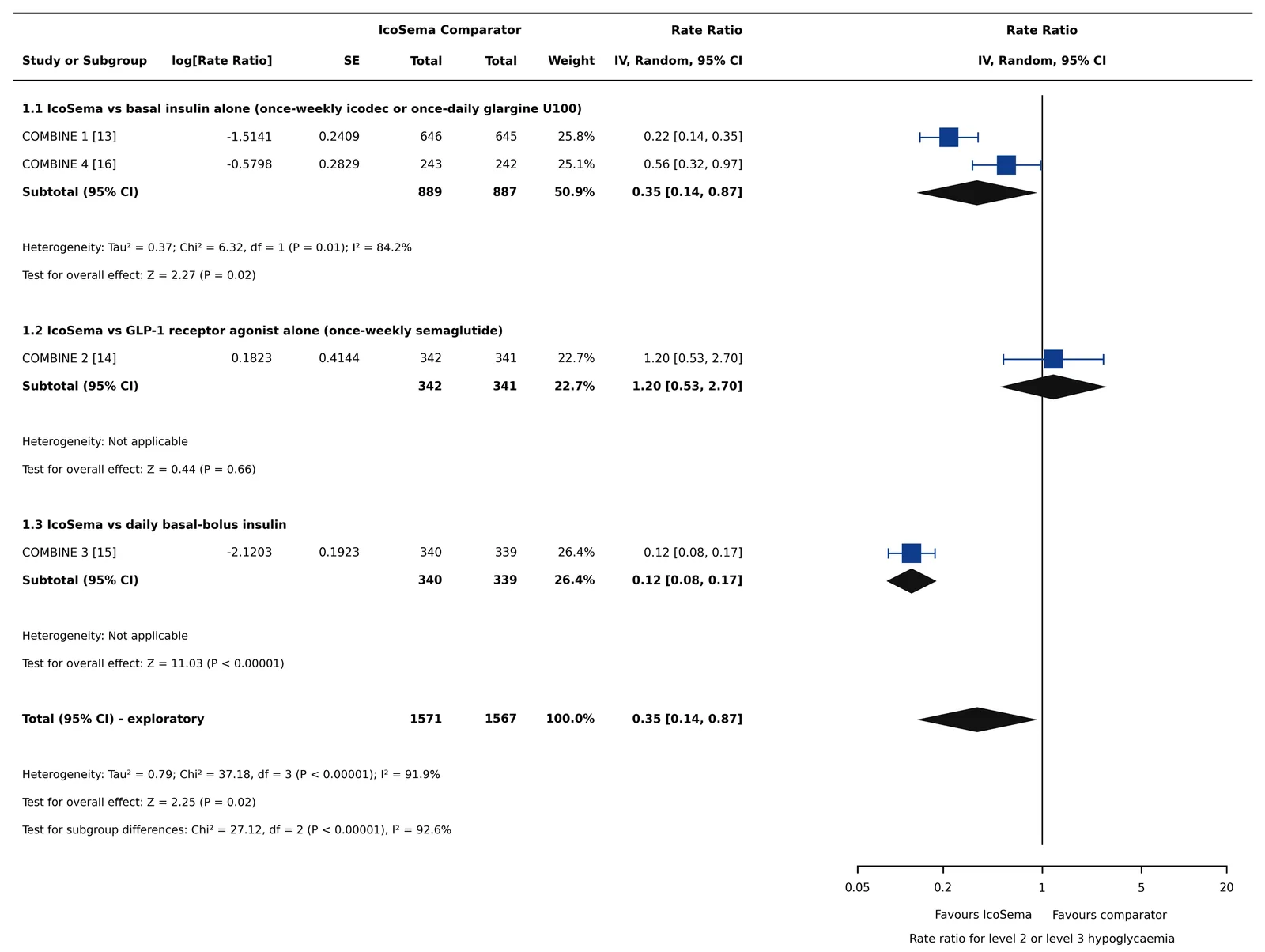
Forest plot of the rate ratio for clinically significant (level 2) or severe (level 3) hypoglycaemia CI: confidence interval; GLP-1: glucagon-like peptide-1; SE: standard error

Other Efficacy and Safety Outcomes

Fasting plasma glucose fell further with IcoSema than with semaglutide (estimated treatment difference −1.07 mmol/L, 95% CI −1.37 to −0.76) [14]. In COMBINE 3, weekly total insulin requirement during weeks 50-52 was 270 U lower with IcoSema than with basal-bolus therapy (95% CI −303 to −236; P < 0.00001), achieved with one injection per week instead of as many as five each day [15]. Continuous glucose monitoring data from COMBINE 1 showed time in range of 75.9% with IcoSema versus 61.9% with icodec [13], and a post hoc analysis of COMBINE 1 and 3 showed lower postprandial glucose increments with IcoSema than with icodec alone, with postprandial control comparable to basal-bolus therapy [33]. Gastrointestinal disorders were the most frequently reported class of adverse event with IcoSema in all four trials, affecting 47% (303/644) of participants in COMBINE 1 and 44% (148/340) in COMBINE 3 [13,15]; arm-level counts for COMBINE 4 are not given in the primary report’s abstract [16]. In COMBINE 2, where both arms contained semaglutide, gastrointestinal events were numerically less frequent with IcoSema than with semaglutide monotherapy (31.4% versus 34.4%), consistent with the lower semaglutide exposure delivered within the fixed-ratio product [14]. Serious adverse events were reported in 9% versus 11% of participants in COMBINE 1 and in 11.1% versus 6.2% in COMBINE 2. An exploratory pool of these two trials gave a risk ratio of 1.21 (95% CI 0.58 to 2.51; I² = 82.7%), an imprecise estimate that excludes neither benefit nor harm and that combines two different comparator classes (Figure 7). Corresponding counts were not extractable from the COMBINE 3 or COMBINE 4 reports available to us and, in keeping with the registered protocol, were not sought from the sponsor. Discontinuation due to adverse events was low and similar between arms in COMBINE 2 (2.3% versus 2.6%), and no treatment-related deaths occurred in COMBINE 1 [13,14].

**Figure 7:**
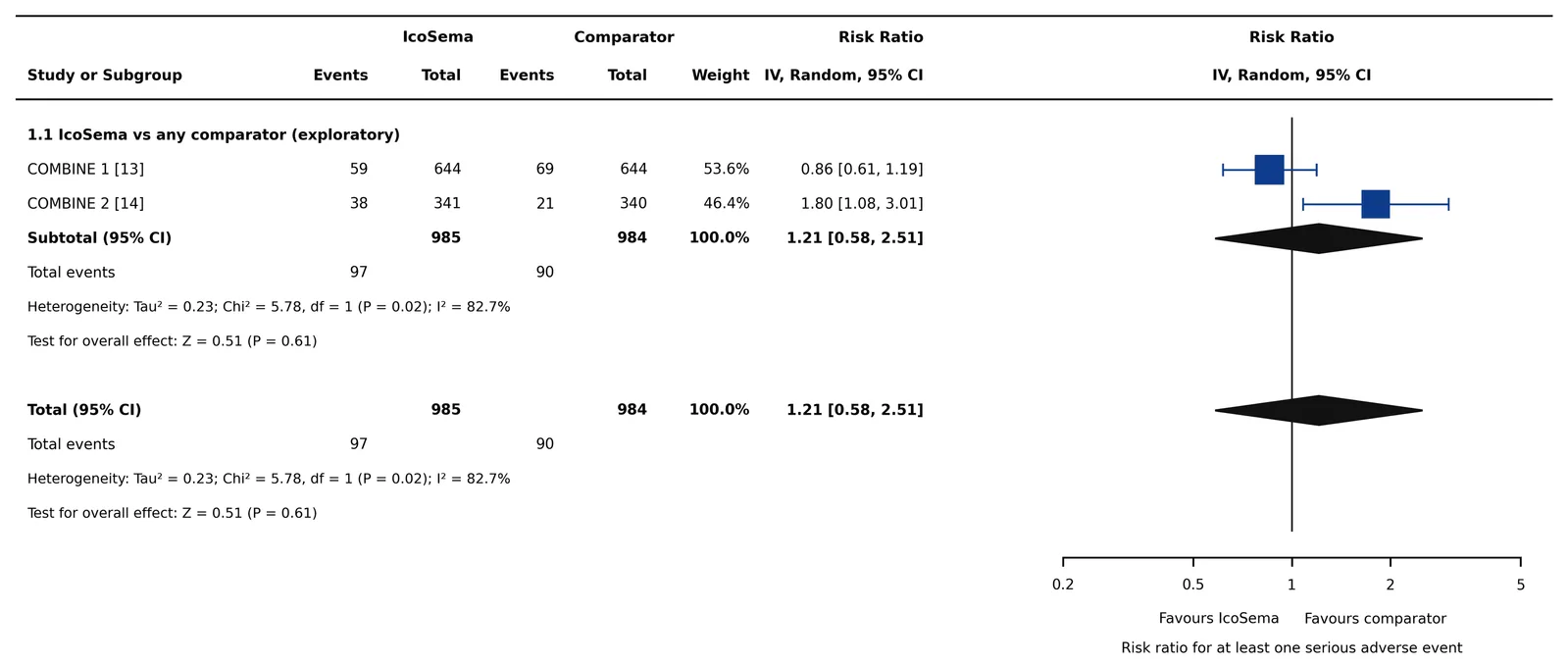
Forest plot of serious adverse events CI: confidence interval; GLP-1: glucagon-like peptide-1

Sensitivity Analyses

Sensitivity analyses are summarised in Table 8. Keeping once-weekly and once-daily basal insulin comparators as separate strata (four strata, each with one trial) did not change any conclusion: the tests for subgroup differences remained significant for HbA1c (χ² = 52.12, df = 3), body weight (χ² = 602.34, df = 3), and hypoglycaemia (χ² = 37.18, df = 3; all P < 0.00001). Fixed-effect estimates for the basal-insulin stratum were similar in magnitude to the random-effects estimates but narrower (HbA1c −0.69 percentage points, 95% CI −0.78 to −0.60; body weight −5.25 kg, 95% CI −5.75 to −4.75; hypoglycaemia rate ratio 0.33, 95% CI 0.23 to 0.47). The Hartung-Knapp adjustment, which uses a t-distribution with one degree of freedom when only two trials contribute, produced intervals too wide to be informative (HbA1c −0.74 percentage points, 95% CI −2.08 to 0.60; body weight −5.15 kg, 95% CI −11.34 to 1.05; hypoglycaemia rate ratio 0.35, 95% CI 0.00 to 131); this is a known property of the method with two studies rather than evidence against the effect, and we report it for completeness. Omitting COMBINE 1, the trial with the imputed standard error, leaves the basal-insulin stratum represented by COMBINE 4 alone (body weight −4.61 kg, 95% CI −5.46 to −3.75) and does not alter the other strata. Leave-one-out analysis of the exploratory all-comparator pool confirmed its instability. Omitting COMBINE 3 moved the HbA1c estimate to −0.64 percentage points (95% CI −0.85 to −0.43; I² = 86%), whereas omitting COMBINE 4 returned it to the previously published value of −0.39 (95% CI −0.71 to −0.07; I² = 95%). For body weight, omitting COMBINE 2, the only trial with a GLP-1 comparator, converted the null pooled result into a large and precise one (−5.64 kg, 95% CI −6.72 to −4.56), although heterogeneity remained considerable (I² = 83%); omitting any other trial left an interval more than 13 kg wide.

**Table 8:** Sensitivity analyses The Hartung-Knapp adjustment with two trials uses a t-distribution with one degree of freedom and is known to produce very wide intervals; it is reported for transparency rather than as the preferred estimate. Estimates for the semaglutide and basal-bolus strata are unaffected by these analyses because each contains a single trial. CI: confidence interval; NA: not assessable (single trial)

Outcome	Analysis	Estimate (95% CI)	I²
HbA1c (%-points)	Basal-insulin stratum, random effects (primary)	−0.74 (−0.95 to −0.53)	62.9%
	Basal-insulin stratum, fixed effect	−0.69 (−0.78 to −0.60)	-
	Basal-insulin stratum, Hartung-Knapp	−0.74 (−2.08 to 0.60)	-
	Basal-insulin stratum, omitting COMBINE 1 (COMBINE 4 only)	−0.88 (−1.12 to −0.63)	NA
	Exploratory pool, all four trials	−0.50 (−0.79 to −0.22)	94.2%
	Exploratory pool, omitting COMBINE 1	−0.45 (−0.83 to −0.06)	94%
	Exploratory pool, omitting COMBINE 2	−0.53 (−0.98 to −0.08)	96%
	Exploratory pool, omitting COMBINE 3	−0.64 (−0.85 to −0.43)	86%
	Exploratory pool, omitting COMBINE 4	−0.39 (−0.71 to −0.07)	95%
	Four separate strata: test for subgroup differences	χ² = 52.12, df = 3, P < 0.00001	94.2%
Body weight (kg)	Basal-insulin stratum, random effects (primary)	−5.15 (−6.10 to −4.19)	69.6%
	Basal-insulin stratum, fixed effect	−5.25 (−5.75 to −4.75)	-
	Basal-insulin stratum, Hartung-Knapp	−5.15 (−11.34 to 1.05)	-
	Basal-insulin stratum, omitting COMBINE 1 (COMBINE 4 only)	−4.61 (−5.46 to −3.75)	NA
	Exploratory pool, all four trials	−3.09 (−8.39 to 2.20)	99.5%
	Exploratory pool, omitting COMBINE 1	−2.26 (−9.43 to 4.91)	100%
	Exploratory pool, omitting COMBINE 2	−5.64 (−6.72 to −4.56)	83%
	Exploratory pool, omitting COMBINE 3	−1.89 (−8.46 to 4.68)	100%
	Exploratory pool, omitting COMBINE 4	−2.59 (−9.66 to 4.48)	100%
	Four separate strata: test for subgroup differences	χ² = 602.34, df = 3, P < 0.00001	99.5%
Level 2/3 hypoglycaemia, rate ratio	Basal-insulin stratum, random effects (primary)	0.35 (0.14 to 0.87)	84.2%
	Basal-insulin stratum, fixed effect	0.33 (0.23 to 0.47)	-
	Basal-insulin stratum, Hartung-Knapp	0.35 (0.00 to 131)	-
	Basal-insulin stratum, omitting COMBINE 1 (COMBINE 4 only)	0.56 (0.32 to 0.97)	NA
	Exploratory pool, all four trials	0.35 (0.14 to 0.87)	91.9%
	Exploratory pool, omitting COMBINE 1	0.42 (0.10 to 1.67)	95%
	Exploratory pool, omitting COMBINE 2	0.24 (0.10 to 0.57)	90%
	Exploratory pool, omitting COMBINE 3	0.51 (0.20 to 1.28)	86%
	Exploratory pool, omitting COMBINE 4	0.30 (0.10 to 0.88)	92%
	Four separate strata: test for subgroup differences	χ² = 37.18, df = 3, P < 0.00001	91.9%

Certainty of Evidence

GRADE ratings are given in Table 9. Certainty for the two-trial basal-insulin estimates was moderate for HbA1c and body weight, downgraded one level for risk of bias arising from the open-label design, and low for hypoglycaemia, downgraded additionally for inconsistency. Certainty for the single-trial estimates versus semaglutide and versus basal-bolus therapy was moderate for HbA1c and body weight and, for hypoglycaemia versus semaglutide, low after an additional downgrade for imprecision; inconsistency could not be assessed for these strata. Certainty for the exploratory all-comparator pools was very low.

**Table 9:** GRADE summary of findings Certainty was rated from a starting point of high for randomised evidence. Single-trial rows are the trial’s own covariate-adjusted estimates presented within the review framework, not pooled estimates. Participant numbers for the serious adverse event row are safety analysis set denominators. CI: confidence interval; MD: mean difference; NA: not assessable (single trial); RaR: rate ratio; RR: risk ratio

Outcome and comparison	Trials (participants)	Effect estimate (95% CI)	I²	Certainty	Reason for downgrading
HbA1c, IcoSema vs. basal insulin alone	2 (1,776)	MD −0.74 %-points (−0.95 to −0.53)	62.9%	⊕⊕⊕◯ moderate	Risk of bias (open-label design)
HbA1c, IcoSema vs. semaglutide 1.0 mg	1 (683)	MD −0.44 %-points (−0.56 to −0.33)	NA	⊕⊕⊕◯ moderate	Risk of bias (open-label design)
HbA1c, IcoSema vs. basal-bolus therapy	1 (679)	MD −0.06 %-points (−0.22 to 0.09)	NA	⊕⊕⊕◯ moderate	Risk of bias (open-label design)
HbA1c, all comparators (exploratory)	4 (3,138)	MD −0.50 %-points (−0.79 to −0.22)	94.2%	⊕◯◯◯ very low	Risk of bias; very serious inconsistency; serious imprecision
Body weight, IcoSema vs. basal insulin alone	2 (1,776)	MD −5.15 kg (−6.10 to −4.19)	69.6%	⊕⊕⊕◯ moderate	Risk of bias (open-label design)
Body weight, IcoSema vs. semaglutide 1.0 mg	1 (683)	MD +4.54 kg (3.84 to 5.23)	NA	⊕⊕⊕◯ moderate	Risk of bias (open-label design)
Body weight, IcoSema vs. basal-bolus therapy	1 (679)	MD −6.72 kg (−7.58 to −5.86)	NA	⊕⊕⊕◯ moderate	Risk of bias (open-label design)
Body weight, all comparators (exploratory)	4 (3,138)	MD −3.09 kg (−8.39 to 2.20)	99.5%	⊕◯◯◯ very low	Risk of bias; very serious inconsistency; serious imprecision
Level 2/3 hypoglycaemia, vs. basal insulin alone	2 (1,776)	RaR 0.35 (0.14 to 0.87)	84.2%	⊕⊕◯◯ low	Risk of bias; serious inconsistency
Level 2/3 hypoglycaemia, vs. semaglutide 1.0 mg	1 (683)	RaR 1.20 (0.53 to 2.69)	NA	⊕⊕◯◯ low	Risk of bias; serious imprecision
Level 2/3 hypoglycaemia, vs. basal-bolus therapy	1 (679)	RaR 0.12 (0.08 to 0.17)	NA	⊕⊕⊕◯ moderate	Risk of bias (open-label design)
Level 2/3 hypoglycaemia, all comparators (exploratory)	4 (3,138)	RaR 0.35 (0.14 to 0.87)	91.9%	⊕◯◯◯ very low	Risk of bias; very serious inconsistency; serious imprecision
Serious adverse events, two trials (exploratory)	2 (1,969)	RR 1.21 (0.58 to 2.51)	82.7%	⊕◯◯◯ very low	Risk of bias; serious inconsistency; serious imprecision

Discussion

Principal Findings

This review brings together the four randomised trials of IcoSema, 3,138 participants in all, and organises them by the clinical question each answers. Against basal insulin alone, the one comparison for which two independent trials now exist, IcoSema lowered HbA1c by 0.74 percentage points, reduced body weight by 5.15 kg, and reduced clinically significant or severe hypoglycaemia by about two-thirds, with moderate certainty for the glycaemic and weight effects. Against daily basal-bolus therapy, a single trial found IcoSema non-inferior for glycaemic control whilst reducing body weight by 6.72 kg, lowering weekly insulin requirement by 270 U, and reducing hypoglycaemia by 88%, with one injection per week rather than as many as five each day. Against semaglutide alone, a single trial found a further 0.44 percentage-point HbA1c reduction and threefold higher odds of reaching HbA1c below 7.0%, at the cost of 4.54 kg of forgone weight loss, with no significant difference in hypoglycaemia. These comparator-specific estimates differ from one another far more than chance would explain, and for body weight they differ in direction. The exploratory pools that ignore comparator tell a much less useful story: a modest HbA1c benefit with I² of 94%, and a body-weight estimate whose CI spans more than 10 kg and includes zero. We have labelled those pools exploratory throughout and do not regard them as estimates of an overall treatment effect.

Interpreting the Heterogeneity

The significant tests for subgroup differences show that between-trial variation is consistent with comparator class being a major source. They do not establish it. In the semaglutide and basal-bolus strata, comparator and trial are one and the same, so any feature of those trials, not only the comparator, could contribute to the difference. The trials also differ in population (insulin-experienced versus insulin-naive), baseline HbA1c (7.9%-8.2% versus 9.5%) and duration (52 versus 40 weeks). The addition of COMBINE 4 illustrates the point: within the basal-insulin stratum, the HbA1c difference was larger and the hypoglycaemia reduction smaller in the insulin-naive, poorly controlled COMBINE 4 population than in COMBINE 1, and the residual I² of 63%-84% within that stratum is plausibly explained by these population differences rather than by anything about IcoSema. What can be said is that the pharmacology predicts the pattern we observed, particularly the reversal of the weight effect, and that pooling across comparators would obscure a difference that clinicians need to see. Whether comparator class is the whole explanation cannot be settled with the present data.

Comparison With Previous Work

Our exploratory four-trial pool for HbA1c (−0.50 percentage points) is more favourable than the three-trial estimate of the earlier pairwise meta-analysis (−0.39 percentage points, 95% CI −0.71 to −0.07, I² = 95%) [17], and omitting COMBINE 4 reproduces that earlier estimate exactly, which serves as an external check on our extraction and computation. The two reviews differ less in arithmetic than in interpretation: the earlier review characterised IcoSema as offering better glycaemic control and lower hypoglycaemia risk than controls on the basis of the pooled estimates, whereas we regard estimates carrying I² above 90% as uninterpretable and present the comparator-specific results instead. A more recent synthesis restricted to COMBINE 1 and 3 excluded COMBINE 2 because its semaglutide comparator addresses a different clinical question, reporting a pooled weight reduction of 6.10 kg and no significant HbA1c effect across those two trials [19]. We agree with that reasoning about COMBINE 2 but take a different route: rather than discard the trial, we present it in its own stratum, so that all randomised evidence is retained and the trade-off it reveals remains visible. A network meta-analysis has positioned IcoSema against once-daily IDegLira, estimating a 0.4 percentage-point HbA1c advantage and a 3.2 kg weight advantage for IcoSema [18], and a synthesis of once-weekly insulins reached compatible conclusions about the class [34]. Read together with the ONWARDS programme [10,11], the picture is coherent: the insulin component drives fasting control, the GLP-1 component contributes postprandial control and the weight benefit, and the once-weekly format removes most of the injection burden.

Clinical Implications

The comparator-specific estimates correspond to distinct clinical decisions and are best considered separately. For a patient already on basal insulin whose HbA1c remains above target, and for an insulin-naive patient in whom basal insulin is about to be started, the evidence from two trials favours IcoSema over basal insulin alone on glucose, weight, and hypoglycaemia. This is the best-supported finding in the review. For a patient facing intensification to basal-bolus therapy, one trial supports IcoSema as an alternative that meets a non-inferiority margin for glycaemic control whilst reducing weight, hypoglycaemia, and injection burden; given the established relationship between injection burden and adherence [4,5], that simplification may matter as much in routine practice as the metabolic differences. For a patient on a GLP-1 receptor agonist alone whose glycaemia is inadequate, one trial shows a trade-off that should be discussed explicitly: better glycaemic control and higher odds of reaching target, against relinquishing about 4.5 kg of weight benefit. Where weight loss is the dominant goal, intensifying the GLP-1 receptor agonist may remain preferable; where glycaemic control is the priority, the single available trial favours IcoSema. A post hoc analysis of COMBINE 1 to 3 suggests that these effects are broadly consistent across kidney and liver function subgroups [35], which supports their applicability to the wider clinical population, though the exclusion of people with severe renal impairment and a body mass index above 40 kg/m² from the trials limits generalisation beyond those bounds.

Limitations

The evidence base is small. Four trials exist, all sponsored, designed, and analysed by the manufacturer, and sponsorship bias cannot be excluded. Two of the three comparator strata contain a single trial, so their estimates are trial-level results presented within a systematic framework; they carry no replication, and inconsistency cannot be assessed for them. Even the basal-insulin stratum contains only two trials, so its heterogeneity statistics are imprecise and, as the Hartung-Knapp analysis shows, alternative estimators give intervals that are uninformatively wide. Comparator and trial are confounded. The significant subgroup tests demonstrate differences between trials but cannot establish that comparator class, rather than population, baseline glycaemia, trial duration, or any other trial-level feature, explains the heterogeneity. We have worded our conclusions accordingly. All four trials were open-label. Masking was not feasible given the difference in injection frequency, and HbA1c and body weight are objective outcomes, but investigator-guided titration and adverse-event reporting could still have been influenced by knowledge of assignment; this was the basis for the RoB 2 judgement of some concerns for every trial. Safety extraction is incomplete. Arm-level counts for serious adverse events in COMBINE 3 and 4, and for gastrointestinal events by preferred term in most trials, were not available in the public record accessible to us, and in keeping with the registered protocol, we did not approach the sponsor; the safety synthesis is therefore narrative where pooling was not possible. Publication bias could not be assessed statistically with fewer than 10 trials; because all trials are registered and sponsor-run, selective non-publication of a completed trial is unlikely, but selective outcome reporting within trials could not be fully excluded. The CI for the body-weight difference in COMBINE 1 was imputed as described, using the more conservative of two available residual standard deviations, and no stratified conclusion depends on it. Follow-up of 40-52 weeks cannot address durability, cardiovascular outcomes, retinopathy progression, or rare adverse events, and aggregate data preclude examination of effect modification by baseline characteristics. The literature is expanding rapidly, and further trials may alter the estimates presented here. Three deviations from the registered protocol are declared. First, the protocol specified assessment with both RoB 1 and RoB 2; we used RoB 2 alone, since RoB 1 is superseded. Second, the protocol stated that certainty would not be assessed; we added the GRADE rating. Third, the protocol specified a search end date of June 30, 2026, and stratification into once-weekly basal insulin, GLP-1 receptor agonist, and basal-bolus comparators; in response to peer review, we updated the search to September 5, 2026, which identified COMBINE 4, and merged its once-daily basal insulin comparator with the once-weekly icodec comparator into a single basal-insulin stratum. That merging decision was made after COMBINE 4 was identified; a four-stratum analysis, which retains the prespecified once-weekly basal-insulin stratum and places COMBINE 4 in a stratum of its own, is reported as a sensitivity analysis and gives the same conclusions.

Future Research

Head-to-head trials against IDegLira and against dual agonists such as tirzepatide would place IcoSema within the fixed-ratio and incretin landscape on direct rather than indirect evidence. Cardiovascular and renal outcome data are absent and will be needed before the combination can be positioned in guidelines for high-risk populations. Longer follow-up is required to establish whether the glycaemic and weight separations observed at 40-52 weeks persist and whether the hypoglycaemia advantage is maintained once titration has stabilised in routine care. Individual participant data would allow effect modification by baseline HbA1c and insulin exposure to be examined properly, which aggregate data cannot support.

## Conclusions

Four randomised trials, 3,138 adults, and 40-52 weeks of treatment support conclusions that differ by comparator. Against basal insulin alone, the comparison supported by two trials, IcoSema lowered HbA1c further, reduced body weight, and caused less hypoglycaemia, with moderate certainty for the glycaemic and weight effects. Against a daily basal-bolus regimen, a single trial found IcoSema non-inferior for glycaemic control whilst reducing body weight, hypoglycaemia, and total insulin dose and replacing daily injections with one a week. Against semaglutide alone, a single trial found that IcoSema lowered HbA1c further but delivered about 4.5 kg less weight loss. Combining these comparisons into a single figure is not informative: the exploratory all-comparator pooled estimates carry I² above 90% and, for body weight, an interval that includes zero and spans more than 10 kg. The evidence is limited by the small number of trials, single-trial strata, common sponsorship, and open-label design, and the findings should be read as comparator-specific and provisional. The practical message is that the value of IcoSema depends on what it would replace, and that patients and clinicians should be shown the estimate for the relevant comparison rather than an average across all of them.

## References

[1] GBD 2021 Diabetes Collaborators (2023). Global, regional, and national burden of diabetes from 1990 to 2021, with projections of prevalence to 2050: a systematic analysis for the Global Burden of Disease Study 2021. Lancet.

[2] Davies MJ, Aroda VR, Collins BS (2022). Management of hyperglycaemia in type 2 diabetes, 2022. A consensus report by the American Diabetes Association (ADA) and the European Association for the Study of Diabetes (EASD). Diabetologia.

[3] American Diabetes Association Professional Practice Committee (2025). 9. Pharmacologic approaches to glycemic treatment: standards of care in diabetes-2025. Diabetes Care.

[4] Peyrot M, Rubin RR, Lauritzen T (2005). Resistance to insulin therapy among patients and providers: results of the cross-national Diabetes Attitudes, Wishes, and Needs (DAWN) study. Diabetes Care.

[5] Polonsky WH, Henry RR (2016). Poor medication adherence in type 2 diabetes: recognizing the scope of the problem and its key contributors. Patient Prefer Adherence.

[6] Nauck MA, Quast DR, Wefers J, Meier JJ (2021). GLP-1 receptor agonists in the treatment of type 2 diabetes-state-of-the-art. Mol Metab.

[7] Hjerpsted JB, Flint A, Brooks A, Axelsen MB, Kvist T, Blundell J (2018). Semaglutide improves postprandial glucose and lipid metabolism, and delays first-hour gastric emptying in subjects with obesity. Diabetes Obes Metab.

[8] Bolli GB, Porcellati F, Lucidi P, Fanelli CG, Perseghin G, Horowitz M, Owens DR (2025). An overview of randomized clinical trials of fixed-ratio combinations of basal insulin plus GLP-1RA (injectable therapy): lessons for advancing therapy in people with type 2 diabetes. Diabetes Obes Metab.

[9] Nishimura E, Pridal L, Glendorf T (2021). Molecular and pharmacological characterization of insulin icodec: a new basal insulin analog designed for once-weekly dosing. BMJ Open Diabetes Res Care.

[10] Rosenstock J, Bain SC, Gowda A (2023). Weekly icodec versus daily glargine U100 in type 2 diabetes without previous insulin. N Engl J Med.

[11] Philis-Tsimikas A, Asong M, Franek E (2023). Switching to once-weekly insulin icodec versus once-daily insulin degludec in individuals with basal insulin-treated type 2 diabetes (ONWARDS 2): a phase 3a, randomised, open label, multicentre, treat-to-target trial. Lancet Diabetes Endocrinol.

[12] Westergaard L, Alifrangis L, Buckley ST (2024). Pharmacokinetic properties of a once-weekly fixed-ratio combination of insulin icodec and semaglutide compared with separate administration of each component in individuals with type 2 diabetes mellitus. Clin Drug Investig.

[13] Mathieu C, Giorgino F, Kim SG (2025). Once-weekly IcoSema versus once-weekly insulin icodec in type 2 diabetes management (COMBINE 1): an open-label, multicentre, treat-to-target, randomised, phase 3a trial. Lancet Diabetes Endocrinol.

[14] Lingvay I, Benamar M, Chen L (2025). Once-weekly IcoSema versus once-weekly semaglutide in adults with type 2 diabetes: the COMBINE 2 randomised clinical trial. Diabetologia.

[15] Billings LK, Andreozzi F, Frederiksen M (2025). Once-weekly IcoSema versus multiple daily insulin injections in type 2 diabetes management (COMBINE 3): an open-label, multicentre, treat-to-target, non-inferiority, randomised, phase 3a trial. Lancet Diabetes Endocrinol.

[16] Ji L, Benamar M, Mohan V (2026). Once-weekly IcoSema versus once-daily insulin glargine U100 in type 2 diabetes management (COMBINE 4): an open-label multicentre, treat-to-target, randomised, phase 3b trial. Lancet Diabetes Endocrinol.

[17] Kamrul-Hasan AB, Chatterjee S, Nagendra L, Patra S, Dutta D, Aljaberi A, Pappachan JM (2025). Efficacy and safety of once-weekly IcoSema in adults with type 2 diabetes: a systematic review and meta-analysis. AACE Endocrinol Diabetes.

[18] Kandalam S, Benamar M, Le Reun C, Rengger L, Gupta P, Nair S (2026). Efficacy and safety of once-weekly IcoSema versus once-daily IDegLira in people with type 2 diabetes: systematic literature review and network meta-analysis. Diabetes Ther.

[19] Alper A, Fagin A, Karthikeyan A (2026). Cardiometabolic outcomes of once-weekly IcoSema in adults with type 2 diabetes: systematic review and meta-analysis of the COMBINE trials. Cardiovasc Diabetol Endocrinol Rep.

[20] Higgins JP, Thomas J, Chandler J, Cumpston M, Li T, Page MJ, Welch VA (2019). Cochrane Handbook for Systematic Reviews of Interventions.

[21] Page MJ, McKenzie JE, Bossuyt PM (2021). The PRISMA 2020 statement: an updated guideline for reporting systematic reviews. BMJ.

[22] Shamseer L, Moher D, Clarke M (2015). Preferred reporting items for systematic review and meta-analysis protocols (PRISMA-P) 2015: elaboration and explanation. BMJ.

[23] International Hypoglycaemia Study Group (2017). Glucose concentrations of less than 3.0 mmol/L (54 mg/dL) should be reported in clinical trials: a joint position statement of the American Diabetes Association and the European Association for the Study of Diabetes. Diabetes Care.

[24] Battelino T, Danne T, Bergenstal RM (2019). Clinical targets for continuous glucose monitoring data interpretation: recommendations from the international consensus on time in range. Diabetes Care.

[25] Sterne JA, Savović J, Page MJ (2019). RoB 2: a revised tool for assessing risk of bias in randomised trials. BMJ.

[26] McGuinness LA, Higgins JP (2021). Risk-of-bias VISualization (robvis): an R package and Shiny web app for visualizing risk-of-bias assessments. Res Synth Methods.

[27] DerSimonian R, Laird N (1986). Meta-analysis in clinical trials. Control Clin Trials.

[28] Higgins JP, Thompson SG (2002). Quantifying heterogeneity in a meta-analysis. Stat Med.

[29] IntHout J, Ioannidis JP, Borm GF (2014). The Hartung-Knapp-Sidik-Jonkman method for random effects meta-analysis is straightforward and considerably outperforms the standard DerSimonian-Laird method. BMC Med Res Methodol.

[30] Egger M, Davey Smith G, Schneider M, Minder C (1997). Bias in meta-analysis detected by a simple, graphical test. BMJ.

[31] Guyatt GH, Oxman AD, Vist GE, Kunz R, Falck-Ytter Y, Alonso-Coello P, Schünemann HJ (2008). GRADE: an emerging consensus on rating quality of evidence and strength of recommendations. BMJ.

[32] Balshem H, Helfand M, Schünemann HJ (2011). GRADE guidelines: 3. Rating the quality of evidence. J Clin Epidemiol.

[33] De Block C, Benamar M, Fu A, Maltesen R, Giorgino F (2026). CGM-derived postprandial glucose with IcoSema versus other insulin regimens: a post hoc analysis of COMBINE 1 and 3. EClinicalMedicine.

[34] Bourron O, Denimal D (2026). Efficacy and safety of once-weekly insulins in type 2 diabetes: a systematic review and meta-analysis. Diabetes Obes Metab.

[35] Ji L, Andersen JD, Benamar M, Ponzani P, Unnikrishnan AG, Vinther S, Rea R (2026). Efficacy and hypoglycaemia outcomes with once-weekly IcoSema versus comparators in individuals with type 2 diabetes by kidney and liver function: a post hoc analysis of the COMBINE 1-3 trials. Diabetes Obes Metab.

